# Targeting the remodeled peritoneal ecosystem: a paradigm shift from tumor cells to the microenvironment in gastric cancer peritoneal metastasis

**DOI:** 10.3389/fimmu.2026.1940799

**Published:** 2026-09-15

**Authors:** Ya-xin Gong, Di Hao, Jia-hao Tan, Lu-ya Li, Jing Bai, Rui Feng

**Affiliations:** Department of Pharmacy, Fourth Hospital of Hebei Medical University, Shijiazhuang, China

**Keywords:** gastric cancer, peritoneal metastasis, targeted therapy, tumor ecology, tumor microenvironment

## Abstract

Peritoneal metastasis (PM) is the most common form of organ-specific metastasis in advanced gastric cancer (GC), and is driven by the dynamic interplay between cancer cells and the tumor microenvironment (TME). This review elaborates on the mechanisms through which cancer cells utilize metabolic reprogramming, mechanical stress, and neural control to shape the microenvironment conducive to tumor growth. It also describes the metastatic cascade of their active dissemination and colonization processes of cancer cells within the peritoneal cavity. Finally, on the basis of these mechanistic insights, we propose a three-step strategy that dismantle physical barriers, blocks nutrient supply, and reverses immune suppression. The aim of this study to transform PM from a treatment challenge into a manageable disease, further improving disease prognosis.

## Graphical Abstract

**Figure:**
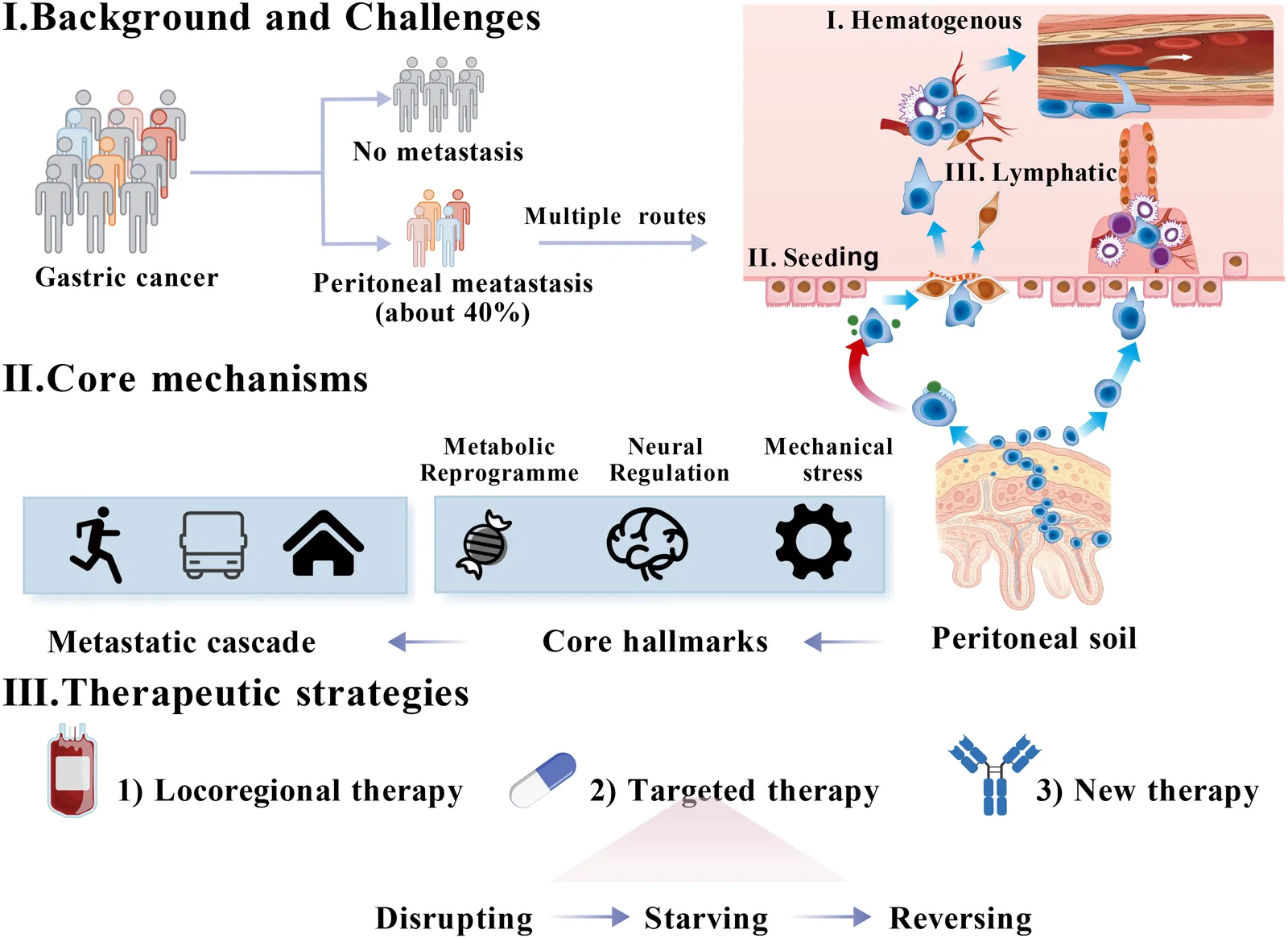

## 1 Introduction

Gastric cancer (GC) is a common malignant tumor of the digestive system (1). Peritoneal metastasis (PM) is the most frequent mode of secondary metastasis in advanced GC, occurring in at least 40% of patients during the disease. The median survival time (MST) of these patients is approximately 7–9 months (2), which underscores an exceptionally poor prognosis.

The mechanisms underlying PM are complex and involve multiple pathways. To provide a clear illustration of the principal routes of GC-PM, three key modes are outlined in **Figure 1**: hematogenous metastasis, seeding metastasis, and lymphatic metastasis. Among these, seeding metastasis is the predominant form of PM formation. This multimodal understanding provides a theoretical foundation for subsequent discussion.

**Figure 1:**
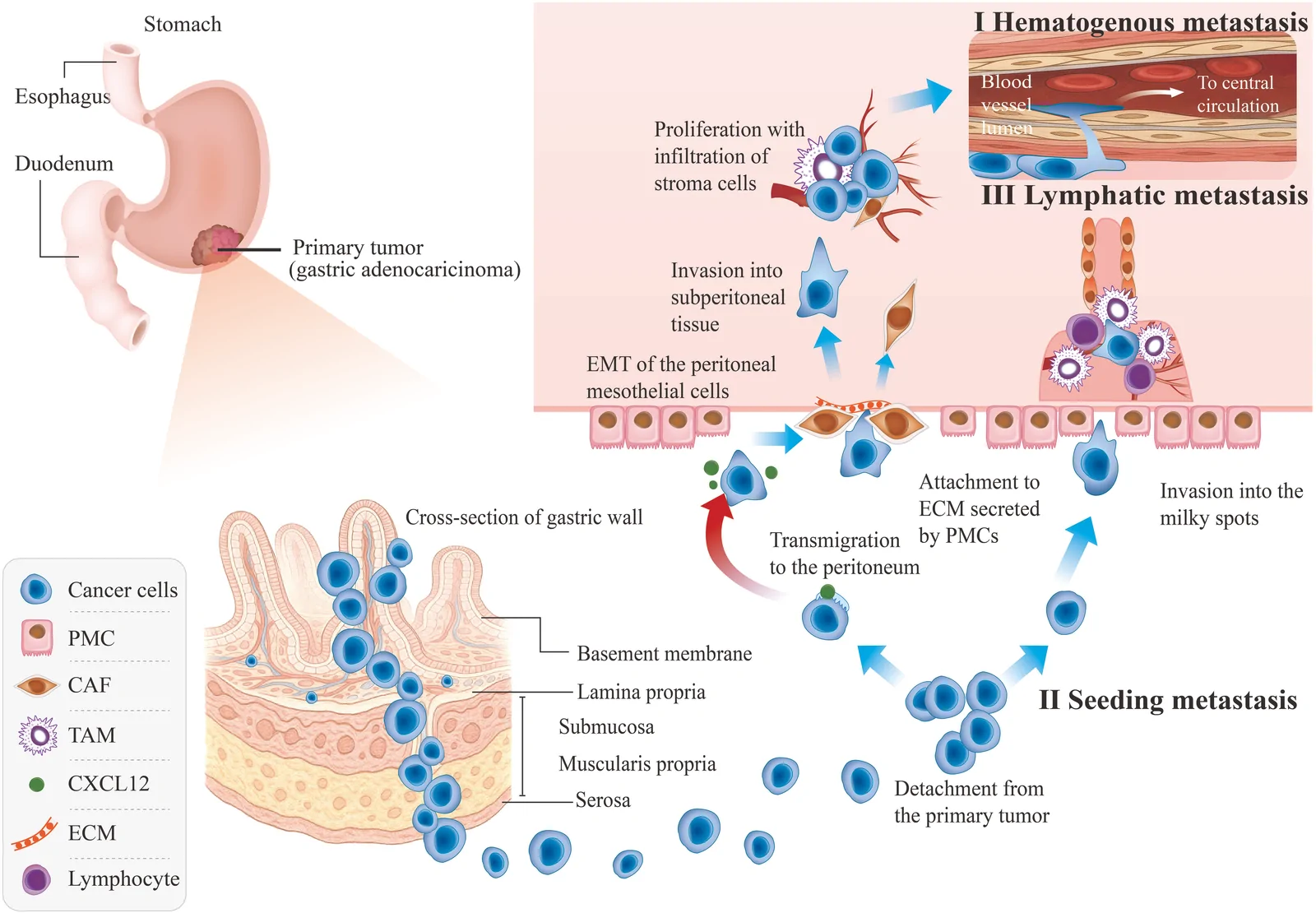
Major routes of GC-PM. I. Hematogenous Metastasis: Tumor cells invade blood vessels, enter the circulation, and disseminate systemically to distant tissues or organs via the bloodstream. II. Seeding Metastasis: Shed cancer cells penetrate the gastric serosa and are released into the peritoneal cavity. They are subsequently implanted on the surfaces of the peritoneum, greater omentum, mesentery, and pelvic organs—much like “seeds”—forming widespread metastatic foci that are often accompanied by malignant ascites. III. Lymphatic Metastasis: Free tumor cells migrate through stomata in the mesothelium and milky spots into the submesothelial initial lymphatic vessels. This schematic illustrates the three principal routes through which cancer cells colonize the peritoneal “soil,” providing a foundation for understanding the physiological peritoneal landscape discussed in Section 2.1.

However, owing to the highly dynamic and complex nature of the GC-PM ecosystem, traditional treatment methods centered on killing cancer cells have limited efficacy. We urgently need new strategies. In this review, we first examine how cancer cells remodel the peritoneum into a prometastatic niche through metabolic reprogramming, mechanical stress, and neural regulation. Building upon this premetastatic remodeling, we then delineate the metastatic cascade within the peritoneal cavity, encompassing detachment, invasion, circulation, extravasation, and colonization. Finally, on the basis of these mechanistic insights, we propose three-step sequential strategy: dismantling physical barriers, blocking nutrient supply, and reversing immunosuppression, aimed at transforming GC-PM from a therapeutic challenge into a more manageable disease.

## 2 The peritoneal “soil”: from physiology to the prometastatic niche

The first step in understanding GC-PM is to recognize how the peritoneum transforms from a normal physiological “soil” into a pathological niche that supports tumor growth. The “seed and soil” theory proposed by Stephen Paget provides a classical framework for understanding cancer metastasis: The spread of peritoneal free cancer cells (PFCCs) is not random but results from interactions between highly metastatic “seeds” and a specific microenvironmental “soil” (3). PM perfectly exemplifies this theory. Using the “seed and soil” theory as a starting point, this section describes how cancer cells hijack and remodel the host’s physiological peritoneal environment after colonization.

### 2.1 Physiological peritoneal landscape

Detached cancer cells first adhere to the peritoneum, a primary target organ for GC metastasis (4). Approximately 34% of patients are diagnosed with PM (5). The peritoneum constitutes a conducive “soil” for GC metastasis, primarily because of two key factors: First, its close anatomical proximity to the stomach facilitates direct spread; second, the rich vascular and lymphatic networks on the peritoneal surface, coupled with circulating ascites, provide a conducive microenvironment and a vehicle for cancer cell dissemination. For instance, CT revealed that gastric adenocarcinoma can spread along vasculature or nerves within subperitoneal ligaments and mesentery (6).

Furthermore, the unique physiological structure of the peritoneum facilitates cancer cell colonization. The peritoneum is divided into parietal and visceral layers, which are continuous with each other and form the peritoneal cavity (7), separated by ligaments, the greater omentum, and mesenteries. Multiple studies have shown that the greater omentum is a preferential site for the metastasis of various types of epithelial malignancies, including GC (8, 9). A large-sample epidemiological study revealed that the liver and greater omentum are the most common metastatic sites, collectively accounting for approximately 76.8% of all metastatic cases (10). Further research has proved that early ovarian tumor (OvCa) actively coordinate tropism for omental metastasis by inducing neutrophil influx and chromatin extrusion in the premetastatic niche (11). The ability of the greater omentum to induce cancer cell colonization is primarily attributed to its immune cell aggregates, known as milky spots, which are rich in high-density vasculature. Milky spots consist mainly of lymphocytes and macrophages (12, 13), which enable preferential cancer cell colonization. Normally, these immune cells clear pathogens and foreign substances from the abdominal cavity. In the tumor environment, they are reprogrammed by tumor-secreted chemokines. One report stated that macrophages in the mesentery of mice promoted the migration and colonization of ovarian cancer cells in the mesentery through the secretion of ligands that interact with the chemokine receptor 1 (CCR1) (14). This finding provides us with an idea for developing new targeted drugs.

Furthermore, the greater omentum is rich in vasculature and adipose tissue, providing an ideal growth environment for cancer cells. Peritoneal adipocytes transfer fatty acids to GC cells, thereby activating the key gene ORAI2 and, ultimately driving the metastasis and colonization (15). Omental adipocytes trigger an invasive phenotype in GC cells through CXCL12-VEGFA signaling, thereby inducing angiogenesis and promoting cancer progression (16). By offering anchoring sites and nutrient supply, the peritoneum plays a crucial role as a highly suitable “soil” in cancer metastasis.

In addition to its macroscopic structure, the integrity of the peritoneal microstructure is closely related to whether cancer cells can be successfully implanted. Microscopically, the peritoneum consists primarily of mesothelial cells and submesothelial connective tissue. Recent studies have shown that impaired mesothelial barrier function renders the underlying peritoneum more susceptible to tumor cell invasion (17). Consistently, GC-derived exosomes can induce mesothelial cell damage, exposing the underlying extracellular matrix (ECM) (18). The ECM is composed of fibrous proteins, proteoglycans, glycosaminoglycans, and glycoproteins and provides structural and signaling support for tumor cell growth and migration. For instance, increased collagen production and cross-linking are associated with increased tumor stiffness and disease progression, whereas fibronectin participates in cell adhesion and migration. The exposed ECM offers an initial landing site for cancer cells, which attach to the peritoneal surface via cell surface receptors such as integrins, thereby facilitating peritoneal metastasis (19). Mesothelial cells within milky spots can also influence cancer cell colonization and metastasis through the expression of adhesion molecules that mediate malignant cell attachment and the secretion of proinflammatory cytokines and chemokines in response to peritoneal infarction. The CCL22-CCR4 axis can promote the localization of free GC cells within milky spots and facilitate their transfer as cell clusters (20). Thus, floating cancer cells acquire a foothold; via interaction between surface adhesion molecules and specific ECM components, they complete the free-to-anchored transition.

### 2.2 Tumor-mediated remodeling of the niche

After establishing an initial foothold in the peritoneum, cancer cells proactively initiate comprehensive reprogramming of the local microenvironment, transforming it into a “pro-tumor ecosystem” that facilitates tumor survival, proliferation, and dissemination while suppressing host immune attack. Cancer cells can reach the subperitoneal mesothelial layer through direct infiltration or lymphatic permeation and secrete various cytokines (21), such as transforming growth factor-beta (TGF-β), vascular endothelial growth factor (VEGF), interleukin-6 (IL-6), and tumor necrosis factor, sometimes in exosomes, thereby establishing a proinflammatory and immunosuppressive microenvironment. Additionally, they can induce mesothelial cells to undergo mesothelial-to-mesenchymal transition (MMT). MMT drives mesothelial cells to contract, lose their epithelial features, and acquire a mesenchymal phenotype, enhancing their invasiveness and migration. A subset of these cells may also transform into cancer-associated fibroblasts (CAFs) (22).

#### 2.2.1 Fostering an immunosuppressive microenvironment

A growing body of evidence indicates that the interaction between CAFs and immune cells can promote immune evasion by tumor cells and drive metastatic progression. Crosstalk between CAFs and tumor-associated macrophages (TAMs) drives resistance to immune checkpoint blockade in GC-PM (23). CAFs also can regulate the epithelial-mesenchymal transition (EMT) process in tumors by promoting the expression of PD-L1, the ligand for the immune checkpoint PD-1 (24). In addition to CAFs, myeloid-derived suppressor cells (MDSCs) are recruited to tumor tissues via the CCL5-CCR5 chemokine axis, which strongly suppresses T-cell function (25). GC cells secrete granulocyte-macrophage colony-stimulating factor (GM-CSF), which upregulates PD-L1 expression in neutrophils through the JAK/STAT3 signaling pathway, thereby inhibiting T-cell proliferation and reducing interferon-gamma (IFN-γ) production (26). Ultimately, these interactions contribute to the formation of an immunosuppressive microenvironment. Through these mechanisms, malignant tumor cells successfully transform from “targets for immune surveillance and elimination” into entities capable of surviving and proliferating uncontrollably within the host.

#### 2.2.2 Dysregulation of angiogenesis and interstitial fluid pressure

In this immunosuppressive microenvironment, tumors evade immune attacks and continue to proliferate. To meet the oxygen and nutrient demands of rapid growth, cancer cells secrete VEGF to promote angiogenesis. VEGF directly acts on endothelial cells, regulates mucin synthesis, and increases vascular permeability (27), thereby increasing the likelihood of cancer cell evasion. However, the distribution of secreted VEGF is typically imbalanced, resulting in the formation of predominantly abnormal and dysfunctional blood vessels (28). These malformed or leaky vessels lead to inadequate perfusion of nutrients and oxygen within the primary tumor site, resulting in the generation of hypoxic, acidic regions and elevated interstitial fluid pressure (IFP) (29). In this high-IFP environment, drug delivery within the tumor relies solely on short-distance passive diffusion, with penetration depth often limited to approximately 100 μm around blood vessels (30). Consequently, the efficacy of systemic chemotherapy is significantly reduced, while the survival probability of cancer cells increases.

#### 2.2.3 Formation of malignant ascites and its impact on the microenvironment

Increased vascular permeability significantly increases the number of escaping cancer cells. PFCCs may obstruct lymphatic vessels, thereby disrupting the balance between peritoneal fluid secretion and drainage (31) and leading to the formation of ascites in the abdominal cavity. Researchers utilized artificial ascites to compress OvCa cells and observed a 20 to 30 fold increase in peritoneal adhesion (32). This process also altered the ultrastructure of the peritoneal mesothelial surface, enabling the transport of mitochondria into OvCa cells. The accumulation of malignant ascites can cause a rapid increase in intraperitoneal pressure (IPP). The resulting compression effect modifies the cytoskeletal structure and induces the phenotypic transformation of cells (33), thereby increasing their adhesion to the peritoneum. Concurrently, the increased transport of mitochondria to cancer cells provides them with a more abundant energy supply (34), which further promotes cancer cell proliferation. Although both GC and OvCa are prone to PM and accompanied by significant ascites at advanced stages, no definitive studies have confirmed that this mechanism applies to GC, highlighting the need for further investigation.

In summary, the progression of peritoneal metastasis is a synergistic process initiated by tumor cells in which the host peritoneal environment actively participates. Tumor cells adeptly exploit and subvert inherent physiological processes of the peritoneum-such as immune defense, injury repair, and fluid exchange to construct a macroscopic, systemic pro-tumor niche within the abdominal cavity. This remodeled peritoneal physiology, characterized by profound immunosuppression, dense fibrosis, and unique fluid dynamics, constitutes the primary barrier to effective treatment.

## 3 Core hallmarks of the pro-metastatic TME

As previously discussed, after cancer cells colonize the peritoneum, they profoundly remodel the host’s microenvironment to establish a malignant niche conducive to their survival and proliferation. To gain a deeper understanding of the mechanisms underlying this niche, the following sections focus on the three core pillars of the tumor microenvironment (TME): metabolic reprogramming, mechanical stress, and neural regulation. As illustrated in **Figure 2**, the TME is a complex ecosystem comprising various cellular and noncellular components, and these three pillars serve as the principal drivers of its malignant progression.

**Figure 2:**
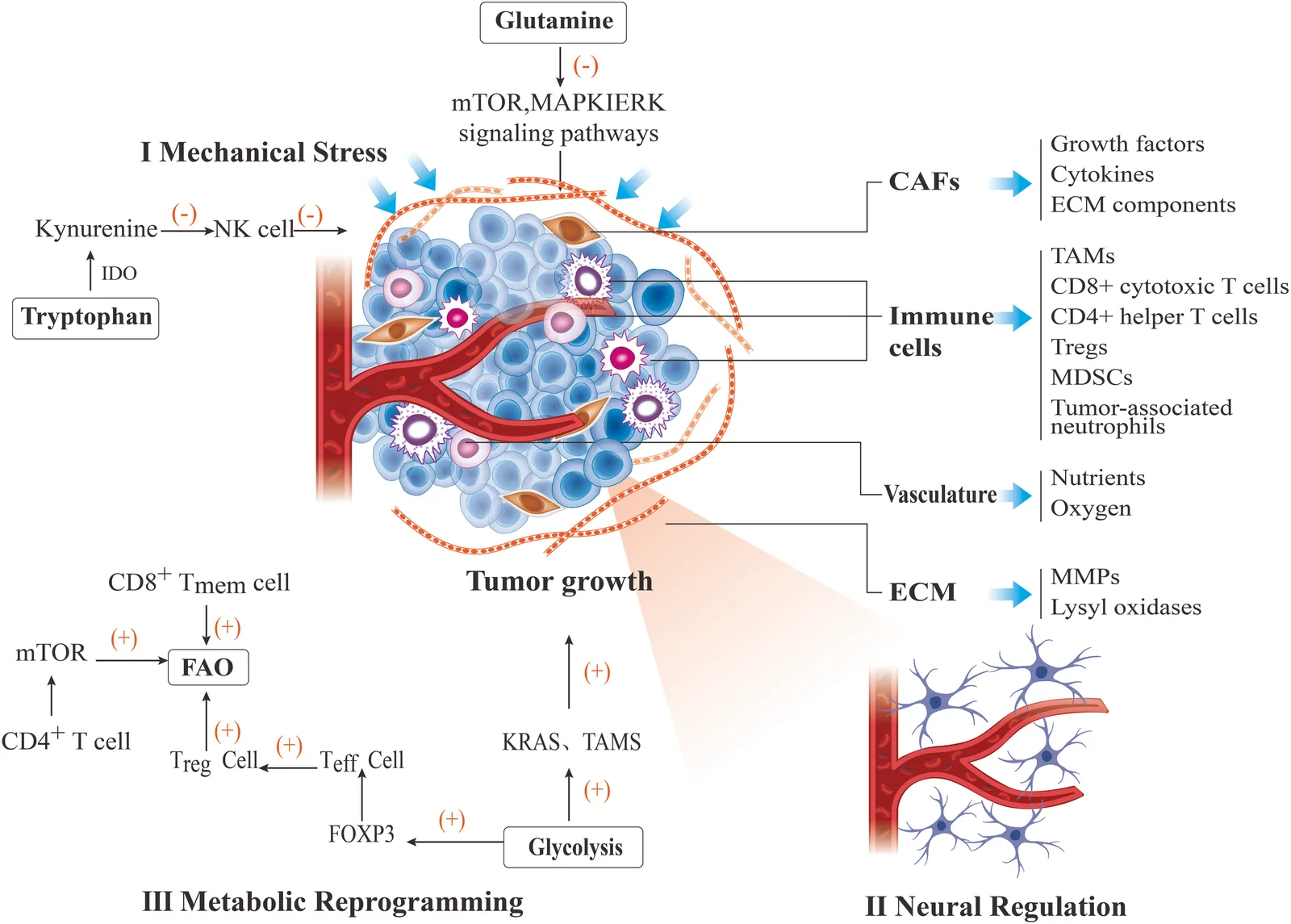
Core components and key mechanisms of the TME in GC-PM. This diagram depicts the complex ecosystem of the TME in GC-PM. Its core cellular and non-cellular components include fibroblasts, immune cells, the vasculature, and the extracellular matrix. Three key mechanisms drive malignant progression and therapeutic resistance in this microenvironment: 1) metabolic reprogramming, characterized by increased glycolysis, dysregulated lipid synthesis, and altered amino acid metabolism; 2) mechanical stress, which contributes to the formation of physical barriers formation and activates protumor mechanosignaling pathways; and 3) neural infiltration, which directly regulates tumor growth and immune activity via neurotransmitters and growth factors. These components and mechanisms are intricately interconnected, collectively forming a dynamic, self-reinforcing ecosystem that supports peritoneal colonization, immune evasion, and sustained tumor progression. The figure not only summarizes the composition of the TME but also, more importantly, positions metabolism, mechanical stress, and neural regulation as central drivers, providing an intuitive logical blueprint for the TME-targeted therapeutic strategies discussed in Section 5.

Tumor cells collaboratively drive TME remodeling and malignant progression through the following multifaceted pathways: first, by establishing metabolic conditions that support energy and biosynthetic demands while promoting immunosuppression; second, by creating physical barriers and activating pro-tumor signaling pathways through mechanical stress; and third, by establishing new growth stimulating networks through neural regulation (35). The interplay among these three factors establishes in a highly malignant TME that promotes tumor progression and therapeutic resistance. Moreover, these findings provide a theoretical foundation for subsequent targeted therapies. For example, mechanical stress can be mitigated using matrix modulators and integrin inhibitors, whereas metabolic reprogramming can be modulated by targeting glycolysis and lipid synthesis. Therefore, elucidating the roles of these three pillars within the TME is fundamental to the design of multidimensional TME-targeted therapeutic strategies.

### 3.1 Metabolic reprogramming

Metabolic dysregulation within the TME has been established as a fundamental hallmark of upper gastrointestinal cancers (36). Through metabolic reprogramming, tumors alter their metabolic pathways to acquire the necessary energy and biosynthetic precursors for survival and proliferation, thereby consolidating a survival advantage within the peritoneal niche. Key manifestations include increased glycolysis, increased competition for glucose and amino acids, abnormal upregulation of protein palmitoylation, and increased fatty acid synthesis (37). Glucose, lipid, and amino acid metabolism are core mechanisms underlying the development of GC-PM. A deeper understanding of the relationship between TME metabolic alterations and cancer progression will facilitate the development of novel therapeutic strategies.

#### 3.1.1 Glucose metabolism

To meet the energy and biosynthetic demands of rapid proliferation, tumor cells alter their metabolic pathways to rely preferentially on glycolysis even under aerobic conditions-a phenomenon known as the Warburg effect (38). This results in the substantial consumption of glucose and amino acids, accompanied by significant lactate production. Lactate can suppress antitumor immune responses through various direct and indirect mechanisms. For instance, L-lactate promotes the proliferation and suppressive function of TGF-β-induced regulatory T cells (Tregs), thereby attenuating tumor immunity (39). The curcumin analog GO-Y022 increases Hk1 expression, promotes glycolysis, generating lactate that acidifies the TME and promotes tumor cell growth (40). Another separate study combined the glucose metabolism-blocking drug metformin with chemotherapeutic agents and find that metformin not only induces apoptosis in GC cells but also counteracts chemotherapy-induced senescence and reduces their clonogenic capacity (41).

The high glycolytic rate of tumor cells depletes most of the glucose within the TME, which leads to glucose starvation in immune cells and subsequently impairs their activity, proliferation, and function. For example, during early tumor stages, TAMs predominantly exhibit an M1 phenotype. These cells demonstrate increased glycolytic and pentose phosphate pathway (PPP) activity to effectively induce inflammatory responses (42). However, as the tumor progresses, most macrophages shift to the M2 phenotype, which relies more heavily on mitochondrial metabolism. Through the tricarboxylic acid (TCA) cycle, increased fatty acid oxidation (FAO), and oxidative phosphorylation (OXPHOS), M2-like TAMs promote immunosuppression and facilitate tumor metastasis (43). M2-like TAMs have been shown to exhibit a heightened capacity for glucose uptake within the TME. This enables these cells to promote O-GlcNAcylation of lysosomal cathepsins, increase the maturation of cathepsin B, degrade proteins in the ECM and basement membrane, and indirectly activate proteases, thereby driving cancer progression and dissemination (44). However, since most of the evidence comes from mouse models with immune function or limited human biopsy samples, simulating immune and metabolic interactions in the highly heterogeneous human TME is impossible. Therefore, further large-scale patient-derived studies are needed to confirm the safety and effectiveness of this approach when it is applied in humans.

#### 3.1.2 Amino acid metabolism

To maintain tumor energy and nitrogen cycling, key α-ketoacids derived from glycolysis and the TCA cycle accept amino groups under the catalysis of transaminases to generate three typical nonessential amino acids: alanine, aspartate, and glutamate (45). Amino acid metabolism is a critical component of metabolic reprogramming in malignant tumor cells. GC cells may alter the uptake, synthesis, and degradation pathways of amino acids to meet their energy and biosynthetic demands during the development of PM. A large-scale prospective cohort study has revealed a significant inverse correlation between total branched-chain amino acid (BCAA) levels and GC risk (P < 0.001), with a more pronounced effect observed in elderly individuals without chronic diseases (46). Additionally, branched-chain amino acid transaminase 1 or 2 promotes BCAA catabolism, which accelerates cell growth and motility (47). The reprogramming of amino acid metabolism is one of the core metabolic features that enables malignant proliferation and PM in GC cells. Through the strategic modulation of amino acid uptake, synthesis, and degradation, GC cells effectively fulfill their specific energy, biosynthesis, redox balance, and signal transduction requirements.

#### 3.1.3 Lipid metabolism

In addition to obtaining energy through glucose and amino acid metabolism, tumor cells can store and generate energy via lipid metabolism. In normal cells, lipid synthesis, lipid droplet storage, and FAO are in a state of dynamic equilibrium. However, the continuous proliferation of cancer cells heightens the demand for the precursors required by these synthesis pathways. GC cells can absorb more exogenous lipids through the CD36 receptor. Increased uptake of exogenous palmitic acid (PA) activates CD36-mediated AKT/GSK-3β/β-catenin signaling, which promotes the migration and invasion of GC cells (48). Adipose tissue is also a major structural component of cell membranes and is classified into two subtypes: white adipose tissue (WAT) and brown adipose tissue. When WAT undergoes cellular remodeling and transitions to a brown-like state, the condition of cancer patients tends to worsen (49). Additionally, adipocytes produce adipokines, which play a critical role in angiogenesis and tumor progression. Lipid metabolites, such as prostaglandins and sphingolipids, can act as signaling molecules to regulate cellular behavior and drive cancer metastasis.

In summary, cancer cells reconfigure the host TME through metabolic reprogramming, linking it to the progression of gastric cancer. However, the clinical translation of metabolic inhibition has not reached the expected level. The current research still has certain limitations. Most studies are based on GC cell lines or mouse models with artificially sufficient nutrition, which cannot simulate the dynamic changes in glucose, oxygen and lipid supplies in the human abdominal cavity. Moreover, mice and humans have different dependencies on metabolites. Therefore, the therapeutic strategy of blocking the tumor nutrient supply remains at the hypothetical level and has not been clinically verified.

### 3.2 Mechanical stress

The TME constitutes a complex “ecosystem”, governed not only by biochemical signals but also by mechanical factors.

#### 3.2.1 Increased ECM stiffness and fibrosis

Mechanical stresses-including solid stress and fluid shear stress, experienced by tumor cells within the TME directly act upon the cells and remodel the physical properties of the tissue by affecting cell stiffness and microstructure (50). These stresses primarily stem from the local compressive effects caused by tumor cell proliferation, excessive ECM deposition, and abnormal angiogenesis. The resulting physical changes not only promote tissue densification and fibrosis but also alter cell morphology and migratory capacity, thereby further driving tumor growth and dissemination (51).

#### 3.2.2 Activation of mechanical signal transduction pathways

Notably, mechanical stress can directly regulate metabolic reprogramming in tumor cells through mechanotransduction pathways. For instance, activation of the YAP/TAZ signaling pathway can upregulate genes related to glycolysis and glutamine metabolism, promote ECM remodeling, and provide the energy and biosynthetic support necessary for malignant tumor progression (52).

During tumor progression, increased matrix stiffness couples with the FAK-RHOA-YAP/TAZ signaling axis to polarize macrophages toward an immunosuppressive M2 phenotype and impair antigen presentation by dendritic cells (DCs) (53). M2 macrophages suppress CD8^+^ T-cell function through the secretion of IL-10 and TGF-β while recruiting Tregs, thereby establishing an immunosuppressive niche (54). Mechanistically, cells sense ECM stiffness and mechanical stress via structures such as integrins and focal adhesions, which activate key cellular signaling pathways. For instance, increased ECM stiffness can upregulate PD-L1 expression in CD8^+^ T cells through the Hippo pathway to promote T-cell exhaustion (55). Activation of the YAP/TAZ signaling pathway upregulates the expression of EMT-related genes, and EMT-associated transcription factors coordinate the release of PD-L1-rich exosomes, thereby disrupting immune surveillance (56) and enhancing the metastatic potential of tumor cells.

#### 3.2.3 Physical barriers impede drug delivery and immune cells

Host tissues exert counteractive compressive forces in response to rapid tumor proliferation, which leads to the accumulation of mechanical stress within the tumor. Increased compression of tumor tissues compacts blood and lymphatic vessels, resulting in a hypoxic TME. This hypoxic environment further accelerates tumor progression by inducing CSC phenotypes, EMT, angiogenesis, inflammation, fibrosis, and immunosuppression (57). To supply oxygen for its own rapid growth, the tumor induces the formation of new blood vessels. However, tumor-induced vessels are immature, highly permeable, and prone to leakage, allowing substantial fluid to enter the extracellular space and uniformly increasing interstitial pressure, which can flush therapeutic agents out of the tumor region (58). The dense ECM and high-pressure environment also impede the infiltration of immune cells, such as cytotoxic T cells, into the tumor core, preventing them from effectively recognizing and eliminating cancer cells and further undermining immune surveillance.

Similarly, although the aforementioned mechanical conduction pathways may promote cancer progression biologically, caution is necessary in their clinical application. Given the complex abdominal environment of the human body and the heterogeneity of tumors, it is still necessary to identify relevant biomarkers, distinguish patients who may benefit, and conduct clinical trials to determine the safety and efficacy of these biomarkers.

### 3.3 Neural regulation

Emerging research suggests that sympathetic and sensory nerves directly influence tumor cells via neurotransmitters that act on adrenergic receptors. This activation triggers signaling pathways including cAMP-PKA, MAPK, and PI3K-AKT, leading to the upregulation of key metabolic enzymes involved in glycolysis and lipid synthesis, thereby facilitating metabolic reprogramming. Neural regulation therefore plays a significant role in tumor progression (59).

#### 3.3.1 Tumor-related neurogenesis

Although neural regulation supports tumor growth by influencing energy availability through multiple mechanisms, it has often been overlooked in therapeutic strategies. Historically, tumors were viewed merely as rapidly proliferating clusters of cancer cells. Despite the discovery of nerve fibers in several cancer types as early as the 1900s, the role of the nervous system in solid tumor progression has been gradually revealed only in recent years (60). In 2001, researchers reported that mouse neurons co-cultured with prostate cancer cells actively extended their neurites toward cancer cells, ultimately stimulating cancer cell growth (61). A 2013 study further revealed that prostate tumors are innervated by both adrenergic sympathetic and cholinergic nerves and that ablation of these nerves inhibits tumor development *in vivo (*62). Researchers have increasingly recognized that tumor tissues are not independent malignant systems; rather, they engage in complex and active communication with the host, particularly the nervous system.

#### 3.3.2 Regulation of tumor growth and immunity

Nerve fibers present in tumor tissues actively participate in tumor progression—a process known as tumor innervation. These nerves primarily originate from the autonomic and sensory nervous systems. Different types of nerves influence malignant progression through distinct signaling pathways. Vagus nerve innervation promotes gastric tumorigenesis via M3 receptor-mediated Wnt signaling in stem cells (63). GC cells attract CGRP^+^ expressing sensory neurons through the NGF-TRK pathway, and upon activation, these neurons promote GC growth and metastasis via the CGRP-RAMP1 axis (64).

In addition to influencing signaling pathways, the activated nervous system is crucial in modulating immune responses and angiogenesis. Acetylcholine (Ach) from the vagus nerve or local cholinergic nerves can bind to muscarinic receptors (CHRM1) on GC cells, activating G q-coupled signaling and ultimately stimulating the PI3K/Akt/mTOR pathway to promote tumor cell proliferation (65). The M3 muscarinic acetylcholine receptor (mAChR), which is specific to parasympathetic signaling, is overexpressed in GC tissues. Activation of this receptor stimulates the Hippo-YAP and Wnt pathways to promote tumor cell proliferation and stemness. Notably, M3 expression is positively correlated with poorly differentiated (G3) tumors (66). The concept of tumor innervation highlights the pivotal role of the nervous system in tumor initiation, progression, and metastasis, challenging the traditional view that nerves are primarily confined to the mediation of sensory, motor, and autonomic functions in normal organs. Accumulating evidence suggests that tumor tissues are densely innervated and can actively regulate tumor progression through the release of various neurotransmitters and neuromodulators. In conclusion, neural regulation plays a nonnegligible role in GC-PM and presents novel targets for therapeutic intervention.

Together with metabolic and mechanical reprogramming, neural infiltration completes a triad of microenvironmental adaptations that collectively stimulate the metastatic cascade.

Although some studies have demonstrated a correlation between the nervous system and cancer cells, in-depth research in this field is lacking, and some speculations and uncertainties still exist. Researchers have not yet clearly determined whether metastatic cancer foci are also under neural control and whether various neurotransmitters with multiple functions can exert the expected effects. In the future, chemical staining can be used to specifically assess neural control status and can be combined with the patient’s condition for analysis, to facilitate the translation of mechanistic findings into clinical practice.

## 4 The metastatic cascade within the peritoneal cavity

Once PFCCs successfully establish a pro-survival niche, they do not halt their progression. To achieve systemic dissemination and distant colonization, they exploit the highly adaptive and supportive TME, which is collectively shaped by metabolic reprogramming, mechanical stress, and neural innervation (as detailed in the preceding chapter), to initiate a series of tightly coordinated biological programs, known as the metastatic cascade. This cascade encompasses complex interactions between free cancer cells and the metastatic microenvironment, resulting in distinct nonrandomness and organ tropism. Beginning with the detachment of cancer cells, this chapter will systematically elaborate on complete process of invasion, circulation, extravasation, and colonization, while revealing how various components within the TME cooperate to drive this progression.

### 4.1 Cancer cell invasion and intravasation

The metastatic cascade commences with the invasion of adjacent tissues by tumor cells that have become detached from the primary lesion. This invasive process typically involves the occurrence of EMT, which cancer cells lose their epithelial characteristics and acquire mesenchymal features, leading to increased migratory and invasive capabilities (67). Concurrently, tumor cells degrade the ECM, which thereby creating dissemination pathways and enabling access to the blood or lymphatic system to facilitate hematogenous or lymphatic metastasis. On the basis of histopathological diagnostic findings, it can be inferred that cancer cells may infiltrate submucosal lymphatic vessels, subsequently metastasizing to the serosa or regional lymphatics to form secondary tumors, and eventually spreading to the peritoneum. Alternatively, they may directly enter the abdominal cavity via metastatic lymph nodes or peritoneal lymphatics, and ultimately even access the bloodstream through the thoracic duct, thus leading to hematogenous metastasis.

Various cellular and noncellular components within the TME play crucial roles in the process of cancer metastasis. Recent studies have indicated that CAFs are among the most abundant cellular components in many solid tumors. Since they differentiate from their precursors multiple growth factors, cytokines, chemokines, and key signaling molecules, CAFs significantly increase tumor metastasis (68). They drive metastasis by remodeling the ECM via the secretion of matrix metalloproteinases (MMPs) that degrade the ECM and reduce intercellular adhesion. Helicobacter pylori activate CAFs to drive the differentiation of gastric epithelial cells associated with CSCs in a manner partially dependent on TGF-β signaling, thereby promoting tumorigenesis (69). TGF-β inhibitors reduce spheroid colony formation and CSC marker expression in GC cells cocultured with CAFs, confirming the involvement of TGF-β signaling in CAF-induced stemness (70). Additionally, CAFs produce high levels of platelet-derived growth factor C (PDGFC), which drives the growth and metastasis of gastrointestinal stromal tumors through paracrine signaling networks (71), underscoring the pivotal role of CAFs in cancer cell dissemination. By selectively targeting relevant pathways to modulate CAF activity, the likelihood of cancer cell metastasis may be reduced, which could potentially curb disease progression from the outset of the metastatic cascade.

Like CAFs, tumor-associated neutrophils (TANs) and TAMs can degrade the ECM by secreting MMPs and factors(such as elastase and cathepsins), thereby facilitating ECM remodeling and increasing the likelihood of tumor invasion. In a polyomavirus middle T antigen-overexpressing breast cancer model, TAMs induce angiogenesis via the secretion of VEGFA and increased vascular density, both of which promote tumor cell intravasation. In animal studies, researchers have reported that neutrophils can interact with cancer cells, thereby promoting their proliferation, angiogenesis, and metastasis to distant sites (72).

Unlike traditional therapeutic approaches that focus on the direct killing of tumor cells, alternative strategies leverage the plasticity of TANs and TAMs to reprogram them into antitumor phenotypes. For instance, in the TME of many solid tumors, upregulation of the CSF-1/CSF-1R signaling pathway drives the polarization of TAMs toward the M2 phenotype, promoting angiogenesis and matrix remodeling (73). The use of small-molecule inhibitors or antibodies targeting CSF-1R can deplete TAMs and shift the remaining cells toward the antitumor M1 phenotype. Several CSF-1R inhibitors have already entered clinical trials (74). Alternatively, therapeutic strategies may directly block the features used by TAMs/TANs to aid tumor metastasis, such as inhibiting MMPs to reduce ECM degradation, targeting VEGF to suppress angiogenesis, or simultaneously disrupting cell adhesion and extravasation to prevent the “hitchhiking” behavior of cancer cells.

After local invasion of the primary tumor site is complete, some cancer cells can enter the circulatory system, initiating distant dissemination throughout the body. This stage is often metaphorically described as the “seeding” phase in the “seed and soil” hypothesis: detached tumor cells, akin to “seeds,” are carried throughout the body via the “rivers” of blood or lymphatic fluid.

### 4.2 Cancer cell circulation

Following invasion, cancer cells enter the bloodstream or lymphatic system, extravasate through capillary beds, and form metastatic clones in distant organs. Tumor cells that enter the circulatory system are referred to as circulating tumor cells (CTCs), which play a crucial role in cancer metastasis (75).

However, the circulatory system presents a hostile environment, and the dissemination of tumor cells faces numerous obstacles, such as anoikis, shear stress from blood flow, and immune system attacks. Anoikis is recognized as a hallmark of cancer. Its function is to prevent cell migration and inappropriate proliferation in abnormal locations (76). To detach from the primary lesion, tumor cells must acquire mechanisms to resist cell death, persist in the circulation, and facilitate metastatic seeding. Melanoma cells, for example, co-opt EMT-like pathways to drive metastatic progression through resistance to anoikis, supporting this notion (77). Although CTCs face significant challenges during dissemination in the circulatory system, most of these cells are positive for Ki67, which indicates that they are in an active proliferative state (78). CTCs have evolved strategies to evade immune surveillance, enabling their survival and eventual metastasis, and a growing body of research is elucidating these underlying mechanisms.

NK cells and monocyte subsets continuously patrol the circulatory system to eliminate CTCs. Consequently, only few CTCs manage to form proliferative metastatic foci in distant organs. However, platelets can assist CTCs in evading immune surveillance by forming a physical barrier, transferring major histocompatibility complex (MHC) class I molecules to the CTC surface, and adhering to them (79). In addition to immune-mediated clearance, the strong mechanical shear forces generated by high-speed blood flow also pose significant challenges to CTC dissemination. However, in-depth studies have shown that under such high shear stress, CTCs undergo mechanical stiffening, which may instead increase their resistance to shear forces and improve their tolerance to immune cell-mediated death (80).

### 4.3 Extravasation and dissemination of cancer cells in distant tissues

Surviving CTCs must undergo multiple steps to establish metastatic lesions in distant organs, including adhesion to endothelial cells, trans endothelial migration, degradation of the ECM, and invasion into the parenchyma. The interaction between CTCs and endothelial cells within the metastatic niche is critical. Upon reaching capillaries, adhesion molecules on the CTC surface bind to corresponding receptors on vascular endothelial cells, which facilitates their adhesion to the vessel wall. Intercellular adhesion molecule-1 (ICAM1) promotes the formation of CTC clusters in breast cancer lung metastasis, facilitating transendothelial migration and lung colonization by tumor cells (81). E-selectin on the vascular endothelium also facilitates the retention of CTCs within capillary beds (82). Beyond endothelial interactions, the tumor cell cluster-coagulation-adhesion axis involving CTCs and platelets plays a significant role in tumor metastasis. Cancer cells can express various procoagulant proteins, such as tissue factor (TF), which activate the coagulation system and trigger active fibrin formation on platelet surfaces (83). Through mutual interaction, a fibrin-platelet sheath is formed, which protects the cell clusters from anoikis and immune attacks. Via platelet bridging and direct interactions, the encapsulated cell clusters roll, decelerate, and firmly adhere to the microvascular endothelium of distant organs.

After successful adhesion, CTCs must traverse the endothelial cell layer and the underlying basement membrane to enter the parenchymal tissue of an organ, a process known as trans endothelial migration. First, CTCs secrete signaling molecules that induce endothelial cell retraction, which loosen intercellular junctions and create gaps. VEGF derived from cancer cells is a key regulator of vascular permeability and endothelial remodeling. Studies have demonstrated that in lipid keratopathy, VEGF stimulates endothelial cell proliferation and migration, thereby promoting angiogenesis and increasing vascular permeability (84). Once gaps form between endothelial cells, CTCs employ amoeboid-like movement to “squeeze” through these narrow spaces, akin to squeezing toothpaste (85). After they successfully cross the endothelial barrier, CTCs encounter the dense protein network of the basement membrane. Tumor cells can secrete MMPs to degrade the ECM, which generates space for cancer cell migration and proliferation.

In summary, the metastasis cascade involves intricate interactions among various cellular and noncellular components within the microenvironment. To systematically outline the molecular targets for intervention in the GC–PM metastatic cascade, **Table 1** categorizes key molecules according to the stages of metastasis: detachment, transport, colonization, invasion, proliferation, and microenvironment remodeling. Its functions, associations with PM, and therapeutic targeting potential are detailed, form a concise “molecular feature-treatment strategy” pairing framework. Clinicians and researchers can prioritize combination therapies targeting specific stages on the basis of disease progression in patients. Future clinical practice could leverage multigene profiling or proteomic analysis of tumor tissues to select individualized treatment regimens with the greatest potential benefit for patients. In summary, the GC–PM cascade involves synergistic interactions across multiple stages and molecules, providing a theoretical foundation for the development of stage-specific therapeutic strategies.

**Table 1:** Key molecular players in the gastric cancer peritoneal metastasis cascade.

Metastatic cascade stage	Molecule	Main functions/mechanisms	Associated with PM	Targeted therapy/potential	Reference
1. Detachment	E-cadherin	Mediates interepithelial adhesion	Downregulation promotes EMT, increasing cell survival, invasion, and migration	Restoration of expression may inhibit progression.	(149)
	EMT transcription factor SNAIL2	As a core regulator of epithelial-mesenchymal transition, SNAIL2 drives acquisition of invasive, drug resistant, and stem like phenotypes, promoting PM.	Drive acquisition of invasive, drug-resistant, and stem-like phenotypes, promoting PM.	SNAIL has been implicated in EMT regulation and correlates with poor prognosis in GC patients.	(150)
	Matrix metalloproteinases (MMPs)	Key proteases that degrade the ECM and basement membrane	Disrupt peritoneal integrity, facilitating cancer cell invasion and implantation.	MMP inhibitors are under investigation as novel biomarkers and therapeutics.	(151)
2. Transport	c-MET/HGF	Receptor-ligand pair that enhances motility and ECM degradation	Stimulates proliferation, invasion and angiogenesis and protects against apoptosis.	Interfering with the binding of HGF to c-MET or inhibiting the dimerization of c-MET can achieve tumor suppression.	(152)
	HER2	Transmembrane tyrosine kinase receptor	Poor overall prognosis in HER2+ GC; association with PM remains unclear.	Trastuzumab is effective but limited in PM settings.	(153)
	IL-6/STAT3	Key inflammatory signaling axis	Activation promotes tumor cell survival, proliferation, angiogenesis, and chemoresistance.	Inhibition may reverse acquired drug resistance.	(154)
	Wnt/β-catenin	Pathway that regulates cell fate and stemness	Induces EMT and chemoresistance.	LncRNA CHAF1B-2 activates the Wnt/β-catenin pathway and promotes GC progression	(155)
	CXCL12/CXCR4	Chemokine axis that direct cell migration	Peritoneal CXCL12 attracts CXCR4+ cancer cells, guiding colonization.	CXCL12 can serve as a potential therapeutic target and biomarker	(156)
3. Colonization	Integrins	Cell adhesion receptors	Increased tumor-ECM adhesion, regulate endothelial barrier, and promote migration/invasion via TGF-β induction.	Potential diagnostic biomarker and therapeutic target	(157)
	CD44	Cell surface adhesion molecule	Promotes proliferation, invasion, and metastasis of GC.	Potential prognostic marker and immunotherapy target.	(158)
	FN1	Major ECM glycoprotein	Mediates stromal-tumor interaction, promoting progression.	Potential prognostic marker	(159)
4. Invasion	uPA/uPAR system	Plasminogen activation system	uPA binding to uPAR activates plasmin, degrading ECM and facilitating invasion.	Expression correlates with PM; may aid in diagnosis.	(160)
5. Proliferation	VEGF/VEGFR	Key regulators of angiogenesis and permeability	Promotes neovascularization, providing oxygen/nutrients and increasing vascular permeability for dissemination.	Anti-VEGF/VEGFR agents (e.g. bevacizumab, ramucirumab) are approved for GC.	(161, 162)
	TGF-β/Smad	Pathway that regulates proliferation, differentiation, migration	Triggers EMT and confers chemoresistance.	Small-molecule TGF-β receptor inhibitors are under clinical evaluation.	(163)
6. Microenvironment remodeling	TAMs	Major cellular component of TME	M2 TAMs promote glycolysis, growth, metastasis, and chemoresistance in GC.	Repolarizing M2 to M1 phenotype is a research hotspot.	(164)
	CAFs	Predominant stromal cell type in TME	Establish a pro-tumor niche by inducing EMT, and promoting immune escape and angiogenesis.	CAF signatures aid in stratifying clinicopathological features and prognosis.	(165)
	PD-L1	Immune checkpoint ligand	Interaction with PD-1 on T cells inhibits anti-tumor immunity; positive expression correlates with PM incidence.	PD-1/PD-L1 blockade has become a promising immunotherapeutic approach in GC.	(166)

## 5 Therapeutic strategies: from local eradication to systemic microenvironment reprogramming

GC–PM is characterized by a high incidence and an extremely poor prognosis. Traditional systemic chemotherapy is often ineffective, primarily because of its limited ability to achieve adequate drug concentrations within the peritoneal cavity-a constraint largely imposed by the blood– peritoneal barrier (BPB). In response to these challenges, therapeutic strategies are evolving from systemic chemotherapy toward more precise and locally intensified approaches.

Currently, cytoreductive surgery (CRS) combined with hyperthermic intraperitoneal chemotherapy (HIPEC) has been established as the standard local-regimen treatment for GC–PM (86). This strategy overcomes physiological barriers to enable high-concentration drug exposure directly within the affected area. Moreover, targeting various components of the TME - such as the vasculature, stromal cells, and metabolic pathway-can fundamentally disrupt the “malignant soil” that sustains metastatic growth. Concurrently, a series of novel therapies, including migration-targeted therapy, photodynamic therapy, and intratumoral immunotherapy, offer more precise and efficient means for direct tumor eradication. Future research will increasingly focus on the integrated and personalized application of these strategies to achieve comprehensive disease control and bring new hope to patients.

### 5.1 Cornerstone: local regional therapy

For patients with GC-PM, the BPB hampers the efficacy of systemic chemotherapy, as drugs do not reach effective concentrations at tumor sites. Additionally, microscopic or extremely small lesions (<1 cm) are difficult to eliminate, often resulting in suboptimal outcomes. The advent of CRS combined with HIPEC has transformed this dilemma, shifting the paradigm from systemic broad-spectrum treatment to locally targeted therapy. This approach has demonstrated promise. In the CYO-CHIP study, compared with CRS alone, CRS plus HIPEC significantly improved OS and recurrence-free survival, which suggests that some patients may achieve a clinical cure with this combined regimen (87). Another phase III trial, GASTRIPEC-II, further confirmed that the CRS plus HIPEC group exhibited significantly better progression-free survival (PFS) and metastasis-free survival (MFS) (88).

#### 5.1.1 Principles and techniques

This strategy relies on CRS to remove macroscopic tumor lesions within the peritoneal cavity, thereby maximally reducing the tumor burden. HIPEC leverages the differential thermal sensitivity of normal tissues and cancer cells by circulating a large volume of heated chemotherapy-containing perfusion fluid at a specific temperature (89). Its core clinical value lies in two aspects: First, the synergistic effects of hyperthermia and chemotherapy enhance drug permeability and cytotoxicity against tumor tissues; second, HIPEC results in “local high-concentration chemotherapy” within the peritoneal cavity, which allows the exposure of PFCCs or microscopic deposits to therapeutic agents. Just like one clinical research about the effectiveness and safety of HIPEC as an adjuvant treatment for stage III GC and reported that the median disease-free survival (DFS) (20.9 months vs. 15.0 months), median OS (42.0 months vs. 31.0 months), and the incidence of peritoneal metastasis (10.5% vs. 21.1%) in the HIPEC group were greater to those in the control group. HIPEC has demonstrated significant therapeutic potential in clinical treatment (90).

HIPEC delivery techniques are categorized into open and closed methods (**Figure 3**). Understanding the configuration for each approach is crucial for optimizing therapeutic efficacy and minimizing complications. After cytoreductive surgery, perfusion can be performed either with the abdomen open or after temporary closure. This involves the placement of catheters for fluid inflow and outflow and a roller pump to drive the recirculation circuit. A heat exchanger maintained the intraperitoneal temperature between 41 °C and 43 °C (91). Once the flow rate and temperature stabilize, the chemoperfusate is added and typically circulated for 90-120 minutes. The choice between open and closed techniques extends beyond procedural details, directly influencing the uniformity of temperature and drug distribution—key determinants of HIPEC efficacy and safety (92).

**Figure 3:**
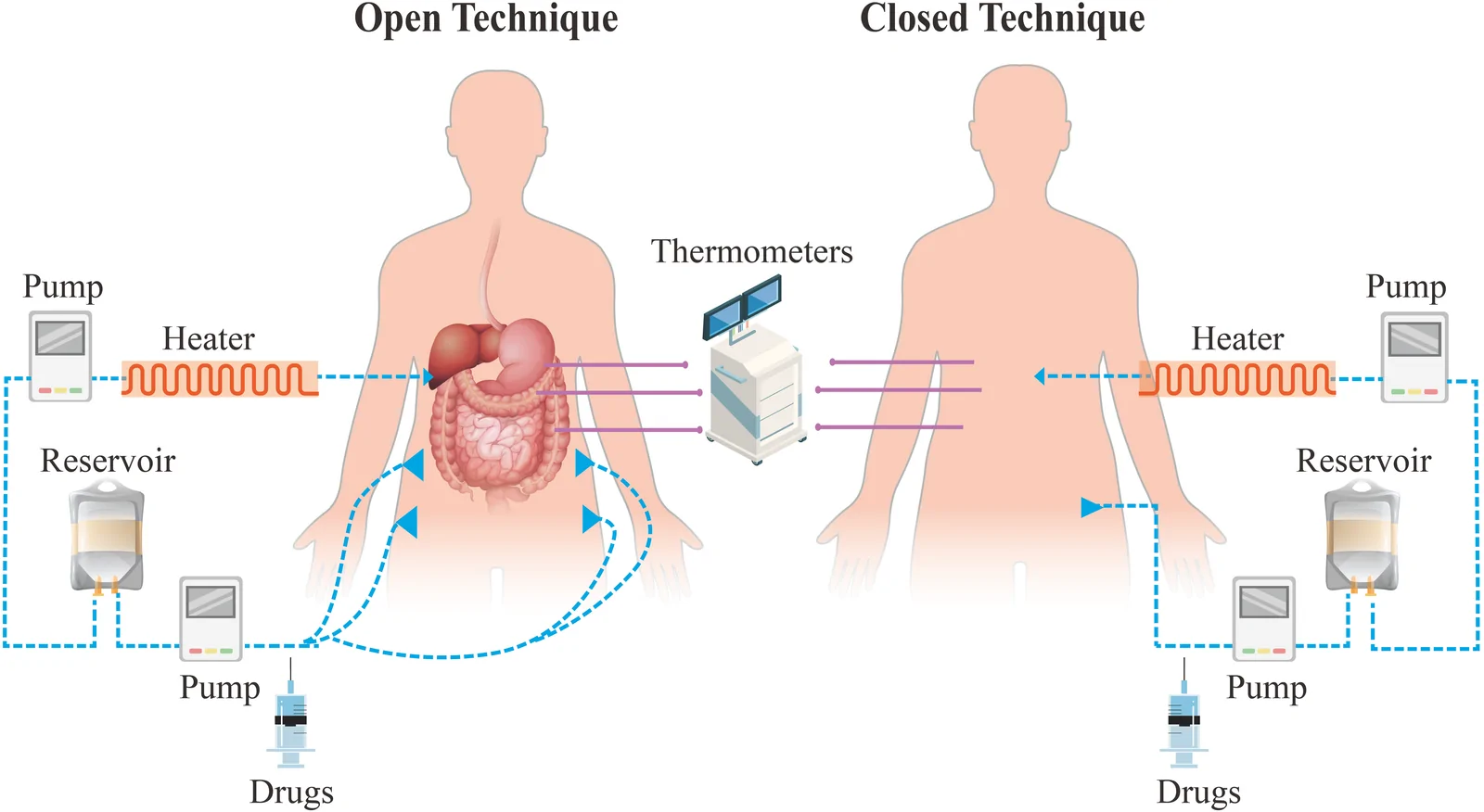
Open versus closed techniques for hyperthermic intraperitoneal chemotherapy (HIPEC) delivery. Both methods require a reservoir, roller pump, heater, and recirculation circuit, with continuous temperature monitoring throughout the procedure. The left panel illustrates the open technique, initially described by Sugarbaker et al., in which the abdominal cavity remains open and the abdominal wall is retracted to expand the perfusion space. Inflow is typically managed via a Tenckhoff catheter, whereas outflow is facilitated by two laterally placed drainage catheters. The right panel depicts the closed technique, where perfusion is performed after temporary or definitive closure of the abdominal cavity, which allows flexible adjustment of the catheter number as needed. The choice of techniques influences the uniformity of temperature and drug distribution, which are critical determinants of HIPEC efficacy and safety.

#### 5.1.2 Fundamental limitations

However, CRS + HIPEC has significant limitations. First, owing to surgical constraints, achieving complete resection is often challenging for diffuse microscopic lesions or retroperitoneal metastases. Second, researchers used fluorescence microscopy to show that intraperitoneally administered paclitaxel (PTX) penetrates only 100 - 200 μm into the tumor surface, which indicates limited druginfiltration into disseminated nodules (93). More fundamentally, this therapy is inherently a “local” clearance strategy and failstoreprogram the tumor-remodeled microenvironment. Consequently, even when macroscopic metastases are removed, residual elements such as the immunosuppressive TME, CAF networks, and proangiogenic signaling can lay the groundwork for recurrence.

In a recent study, researchers used the water-filtered infrared A radiation (WIRA) high-temperature system in combination with immune checkpoint inhibitors (ICIs) for treatment, achieving a 55.6% disease control rate in patients with advanced MSS/pMMR phenotypic gastrointestinal tumors (94).

Although this study did not directly assess the effect of WIRA on the immunosuppressive TME, the clinical results indicate that WIRA may enhance the efficacy of ICIs through thermal effects. This study provides a reference direction for the subsequent development of treatment strategies that reshape the immunosuppressive TME through high-temperature therapy.

### 5.2 Core strategy: targeted attacks on the TME

Despite the improved outcomes with CRS+HIPEC, a significant proportion of patients (e.g., 20.9% in a D2 gastrectomy trial) still develop PM (95). This failure underscores the fundamental limitation of localized cytoreductive strategies: they do not address the remodeled, prometastatic TME itself. Residual cancer cells persist within this conditioned “malignant soil”, which actively supports their survival and regrowth. **Table 2** provides a clear overview of current TME targeting strategies for GC-PM and categorizes TME-targeted agents into four major classes.

**Table 2:** Selected therapeutic agents that target the TME.

Targeted category	Representative drugs/therapies	Target site/mechanism	Drug type	Research stage	Reference
Target the ECM	Andiximab	Inhibits MMP-9 enzymatic activity	Humanized monoclonal antibody	Preclinical	(167)
	Triptonoterpene	Reduces MMP-9/2 protein expression	Natural product extract	Preclinical	(168)
	CAPE	Reduces nuclear β-catenin; inhibits AKT/GSK3β	Synthesizes phenolic acid esters	Preclinical	(169)
	Anithiactin D	Reduces MMP mRNA expression	Natural 2-phenylthiazole	Preclinical	(170)
	Digoxin	Inhibits integrin-mediated α3NaK translocation	Small molecule	Preclinical	(171)
	ATN-161	Antagonizes integrin α5β1 (and αvβ3)	Integrin antagonist peptide	Preclinical	(172)
	Abituzumab	Pan-αv integrin family inhibitor	Humanized monoclonal antibody	Preclinical	(173)
Targeting metabolism	2-DG	Competitively inhibits hexokinase (HK)	Glycolytic inhibitor	Preclinical	(174)
	Metformin	Inhibits proliferation and glycolysis	Biguanide oral hypoglycemic drug	Preclinical	(175)
	Everolimus	mTOR inhibitor; induces apoptosis	mTOR inhibitor	Preclinical	(176)
	Eleutheroside A	Inhibits PI3K/AKT1/mTOR and HIF-1α-glycolysis in MDSCs	Saponin (natural product)	Preclinical	(177)
	Isoliquiritigenin	Blocks glucose uptake and lactate production; suppresses OXPHOS	Flavonoid (natural product)	Preclinical	(178)
	Orlistat	Inhibit lipid synthesis	Lipase inhibitor	Preclinical	(179)
	Piperine	Modulates Acot1-mediated *de novo* adipogenesis	Herbal extract	Preclinical	(180)
	Dehydrocostus lactone (Dehy)	Downregulates ACLY, reducing fatty acid synthesis	Sesquiterpene lactone	Preclinical	(181)
	Nobiletin (NOB)	Reduces *de novo* fatty acid synthesis	Flavonoids	Preclinical	(182)
Anti-angiogenesis	Ramucirumab	Binds and inhibits VEGFR-2	Anti-VEGFR-2 monoclonal antibody	Clinical	(183)
	Apatinib	Selectively inhibits VEGFR-2 intracellular ATP site	Small-molecule VEGFR inhibitor	Clinical	(184)
	Bevacizumab	Neutralizes VEGFA	Anti-VEGF monoclonal antibody	Clinical	(162)
	Fruquintinib	Highly selective VEGFR inhibitor	Small-molecule TKI	Clinical	(185)
	Endostar (recombinant endostatin)	Binds integrins (e.g. α5β1) to, inhibit VEGF signaling and MMPs	Modified recombinant protein	Clinical	(186)
Target cancer-related cells	Qilian Shupi Decoction	Modulates p53/TGF-β signaling, potentially inhibiting EMT	TCM compound	Preclinical	(187)
	Extract of Honghan grass	Repolarizes M2 TAMs to the M1 phenotype by inhibiting TGF-β	TCM extract	Preclinical	(188)
	Dihydroquercetin	Inhibits signaling pathways including TGF-β	Flavonoid (natural compound)	Preclinical	(189)
	Emactuzumab	Inhibits CSF-1R	Monoclonal antibody	Clinical	NCT02323191
	Pexidartinib	Inhibits CSF-1R	Tyrosine kinase inhibitor	Clinical	NCT01525602
	Sildenafil	PDE5 inhibitor; reduces MDSC activity	PDE5 inhibitor	Exploratory application	(190)
	Docetaxel	Induces differentiation of MDSCs	Taxane chemotherapy	Exploratory application	(191)

This table provides an intuitive overview of the current TME-targeting strategies for GC-PM (from natural products to humanized antibodies), and presents some clinical translational value. As shown in the table, traditional Chinese medicine compound preparations and natural product extracts have significant therapeutic potential in targeting the TME. These findings suggest that researchers can combine traditional formulas with modern science to develop more effective active compounds.

Moreover, the table indicates the treatment trend from single-target strategies to multidimensional synergy. The TME is a complex, dynamic and highly plastic ecosystem with multiple components. Future treatment paradigms should be based on the specific TME classification of patients and should involve multitarget synergy treatment plans to achieve systematic reset of the microenvironment.

#### 5.2.1 Disrupting the physical barrier

The TME of GC-PM is characterized by excessive ECM deposition, fibrosis, and elevated IFP, which form a robust physical barrier that supports tumor growth. CAFs are the primary architects of this barrier. They remodel the ECM by secreting MMPs and indirectly contribute to vascular hyperpermeability via VEGF secretion, leading to interstitial fluid accumulation and elevated IFP. In a study, CAFs enhanced the dynamic remodeling of collagen hydrogel structures through dystrophin-mediated contraction and cross-linking, which progressively increased the polymer density to generate mechanical stress and further reinforced the physical barrier (96). Such features not only activate protumor signaling pathways (e.g. YAP/TAZ) but also restrict drug delivery to short-distance passive diffusion, thereby significantly decreasing the efficacy of chemotherapy.

To overcome this physical barrier and improve drug penetration and distribution within tumors, researchers have focused on strategies to remodel the dense, stiff TME, effectively “softening” the tumor. As a major component of solid tumors, CAFs remodel the ECM and secrete growth factors such as TGF-β to promote GC cell survival and proliferation. However, the clinical evidence related to GC-PM that supports this claim is extremely limited. In a clinical trial (NCT02734160) that used the TGF-β inhibitor Galunisertib to treat metastatic pancreatic cancer, the drug demonstrated acceptable safety, but the therapeutic effect was very limited (97). In a preclinical study, eupafolin inhibits GC formation by modulating CAFs through suppression of the IL18/IL18RAP signaling axis (98). Although research on TGF-β receptor inhibitors specifically for GC remains limited, the success of eupafolin highlights the potential of natural product extracts as a source for the development of highly targeted agents. Notably, most drugs targeting the ECM have not yet been applied in clinical practice because of efficacy or safety issues. These findings indicate that although these mechanisms are feasible, in the context of the complex tumor microenvironment, large-scale clinical trials and screening of reliable biomarkers are still necessary to verify their feasibility and safety.

Directly targeting MMPs to interfere with ECM degradation and remodeling is another promising therapeutic approach. For example, Chinese medicine compound Huachansu can induce GC cell apoptosis by inhibiting MMP activity and increasing ROS levels (99). Juglone can target MMP-1 to inhibit its activity and reduce tumor cell invasion and metastasis (100). In addition to CAFs, integrins mediate tumor-ECM interactions. In 482 GC samples, researchers found that at least one of the integrins αvβ3 and αvβ5 was expressed, suggesting that integrin antagonists may have therapeutic value in GC (101). Agents such as cilengitide, ATN-161, and abituzumab have already entered clinical trials.

Despite the mechanistic plausibility of targeting the ECM and CAFs, the clinical translation of these strategies remains nascent. Major obstacles include the functional pleiotropy of CAFs and the lack of specific biomarkers to identify the patients most likely to benefit. Furthermore, the dynamic reciprocity between cancer cells and stromal components complicates the interpretation of preclinical results, as static models fail to capture the spatiotemporal evolution of fibrotic barriers. Future studies should prioritize patient-stratified biomarker discovery and combinatorial approaches that simultaneously target multiple stromal pathways to overcome compensatory feedback loops.

#### 5.2.2 Starving the tumor

After physical barriers are disrupted, deeper penetration into tumor tissue is needed to meet the high oxygen and nutrient demands of rapid proliferation, tumors stimulate the formation of numerous new but abnormal and dysfunctional blood vessels and rapidly acquire energy via mechanisms such as the Warburg effect and increased lipid metabolism. Therefore, antiangiogenic drugs can be employed to normalize the vasculature, alleviate tumor hypoxia and improve drug delivery. Moreover, the inhibition of tumor glycolysis can decrease lactate accumulation, which can help reverse the immunosuppressive effects of the acidic tumor microenvironment.

Theoretically, drugs targeting glycolysis and lipid metabolism hold significant therapeutic potential. With respect to lipid metabolism, the inhibition of sterol o-acyltransferase 1 (SOAT1) and carnitine palmitoyltransferase 1A (CPT1A) in diethylnitrosamine-induced hepatocellular carcinoma (HCC) (102) can inhibit tumor progression. Further research revealed that the SOAT1 inhibitor avasimibe significantly decreases VEGFC expression in GC cells, which suggests that targeting lipid metabolism via SOAT1 inhibition has potential as a therapeutic approach (103). With respect to glycolysis, the natural product Helichrysetin has been shown to regulate GC cell energy metabolism by reducing glycolysis and enhancing mitochondrial oxidative phosphorylation, indicating its potential for modulating metabolic reprogramming (104). Combining platinum-based drugs with G6PD inhibitors can also inhibit tumor cell glycolysis and enhance chemotherapeutic efficacy (105). Although the mechanism is feasible, most drugs targeting glycolysis are still in the preclinical research stage (**Table 2**). We speculate that owing to the limitations of preclinical models, simulating the consumption and dependence of glucose by the human peritoneal environment is environment. Moreover, the numerous and constantly changing targets of glycolysis pose significant challenges for clinical translation.

Research on antiangiogenic drugs has been extensively translated into clinical practice. VEGF and its receptor VEGFR are the most well-established and critical therapeutic targets for these agents (106). Ramucirumab (RAM) and apatinib are representative inhibitors targeting VEGFR-2 and are currently utilized in second-line and third-line chemotherapy regimens, respectively, for advanced GC, thereby offering valuable treatment options for patients at later stages of disease progression (107). Notably, apatinib has been approved in China as a novel therapeutic option for patients with advanced GC who have progressed beyond third-line therapy (108). The performance of apatinib in advanced GC is encouraging, but given that it has not yet been applied in clinical practice is still needed.

Early-phase studies reported promising efficacy of apatinib in advanced GC, with an overall response rate of up to 42% in selected populations (109). However, the subsequent phase IV AHEAD trial, which enrolled a broader patient cohort, reported a more modest median overall survival (OS) of 5.8 months and 87.9% of patients experienced adverse events (110). This discrepancy highlights the challenge of translating promising early-stage results into robust clinical benefits in unselected patient populations.

Furthermore, apatinib exhibits promising efficacy and manageable toxicity in patients with advanced alpha-fetoprotein-producing GC (111). By employing an antiangiogenic “tumor starvation” strategy, apatinib provides renewed hope for improved survival outcomes. Nevertheless, important, antiangiogenic therapies are not universally effective, and significant interindividual variability in treatment response remains a clinical challenge.

A critical caveat in metabolic targeting is the inherent plasticity of cancer cells; it enables rapid switching between glycolytic and oxidative pathways upon pharmacological inhibition. This most likely explains why monotherapy is ineffective in the clinical inhibition of glycolysis. Moreover, the metabolic dependencies of cancer cells differ markedly from those of immune cells within the TME, raising concerns that broad metabolic inhibition may inadvertently compromise antitumor immunity. Future strategies should therefore explore selective inhibitors and rational combination regimens that synchronize metabolic blockade with immunotherapeutic activation, guided by real time metabolic monitoring.

#### 5.2.3 Reversing immunosuppression

Owing to the highly immunosuppressive nature of the TME in GC-PM, which is enriched with TAMs, Tregs, and PD-L1, cytotoxic T cells - become exhausted or fail to infiltrate effectively. However, after these physical barriers are disturbed, vasculature normalized, and hypoxia and acidosis alleviated, immunotherapy efficacy can be maximized. At this stage, efforts can focus on restoring the host’s antitumor immune function.

TAMs are abundant in the gastrointestinal tract, and most are polarized into pro-tumor M2-type TAMs that promote angiogenesis and suppress T-cell responses (112). Modified traditional Chinese medicine can effectively inhibit M2 TAM differentiation by reducing the level of PKM in GC-derived extracellular vesicles(EVs) (113). Carboxymethylated alginate-resiquimod micelles effectively repolarize TAMs from the M2 type to the M1 type *in vivo*, increasing the effects of chemotherapy and immunotherapy and prolonging survival in mice (114). In clinical research, the CSF-1R inhibitor emactuzumab depletes or functionally suppresses M2-type TAMs, which decreases their production of proangiogenic and preinvasive factors and thereby hinders tumor progression (115). Cancer cells can inhibit T-cell function via PD-L1 expression, which can in turn promote disease progression by inducing MDSC expansion or EMT. Drugs targeting PD-L1 are under active development. Simvastatin induces ferroptosis by inhibiting ILF3 in GC cells and enhancing the killing effect of activated CD8^+^ T cells on GC cells, revealing a novel mechanism of action for a drug already on the market (116). In clinical practice, the combination of nivolumab and chemotherapy significantly improves OS and PFS in patients with a PD-L1 CPS ≥5 (117). At this stage, activated T cells can efficiently infiltrate the tumor core and exert potent cytotoxic effects in a relatively favorable microenvironment.

In summary, therapeutic strategies targeting the metastatic TME are progressively shifting toward a synergistic “multimechanism” approach, in which drugs with different modes of action collaborate systematically. This review presents a comprehensive framework that progresses from dismantling physical barriers to reactivating immune responses. Future preclinical research and clinical trial designs should prioritize the exploration of optimal sequences and timing for combining different strategies. Additionally, leveraging multiomics technologies to accurately classify the TME of GC-PM will enable the customization of personalized, systemic treatment regimens for patients. Ultimately, the strategic goal is to transform GC-PM from a therapeutic challenge into a manageable disease.

### 5.3 Novel and emerging modalities

Micrometastases or macrometastases pose a significant threat to cancer patients. Although researchers have made progress in strategies targeting various components of the TME, their efficacy remains limited because of by tumor heterogeneity and the dynamic nature of the TME. In response, a series of more precise and synergistic developing therapies have emerged that aim to overcome existing therapeutic barriers from multiple dimensions. Novel strategies such as migratory therapy, photodynamic therapy (PDT), and targeted intratumoral immunotherapy (ITI) have demonstrated promising therapeutic potential. To provide a horizontal comparison of these emerging approaches, **Table 3** systematically outlines the core mechanisms, targeted TME components, potential advantages, and limitations associated with these three therapeutic strategies.

**Table 3:** Comparison of novel therapeutic modalities for GC peritoneal metastasis.

Therapy category	Photodynamic therapy (PDT)	Migration therapy	Targeted intratumor immunotherapy(ITI)
Core mechanism	Uses photosensitizers selective for tumor tissue. Upon light activation, they generate reactive oxygen species, causing direct tumor damage, vascular injury, and immunogenic cell death.	Aims to prevent cancer cells from leaving the primary site, surviving in circulation, or establishing metastases.	Direct injection of immunostimulatory agents into tumors to achieve high local concentrations, reverse immunosuppression, and induce systemic anti-tumor immunity.
Key TME components targeted	1. Tumor cells (direct cytotoxicity) 2. Tumor vasculature 3. Immune cells (induction of inflammatory response)	1. Cancer cells (via chemokine axes e.g. CXCR4/CXCL12) 2. Extracellular matrix (via MMP inhibition) 3. Circulating tumor cells (CTC clearance)	1. Immunosuppressive cells (TAMs, Tregs, MDSCs) 2. Effector immune cells (T cells, NK cells) 3. Immune checkpoint molecules (PD 1/PD L1)
Potential advantages	Noninvasive, tolerable, can selectively target tumor tissue and induce systemic immune effects.	May reduce metastatic rate and delay onset by intervening early in the dissemination process.	Inhibits local immunosuppression, triggers systemic immunity (“*in situ* vaccine” effect), and significantly reduces systemic toxicity.
Major limitations	Limited light penetration depth; requires complex laparoscopic light delivery systems; limited capacity to remodel established immunosuppressive TME as monotherapy.	High plasticity and redundancy of metastatic pathways; requires multi target combinations.	Variable responses across different lesions; potential for immune related adverse events; technically dependent on accurate intratumoral delivery.
Representative drugs/technologies	5 ALA, Photofrin, Talaporfin, Temoporfin	CXCR4/CXCL12 axis inhibitors, MMP inhibitors, CTC filtration/clearance technologies	Oncolytic viruses (e.g., CF33 based constructs), mRNA vaccines, cytokine formulations, local TLR agonists
Reference	(192)	(193)	(194)

#### 5.3.1 Migration therapy (migration-targeted therapy)

Migration therapy, which aims to prevent tumor cells from migrating from the primary site and seeding at another site, also reduces their metastatic potential and thus represents a promising therapeutic strategy. Rather than seeking to directly eliminate all cancer cells this therapy focuses on preventing these cells from leaving the primary lesion, surviving in circulation, or establishing themselves in new locations. These findings have important implications for preventing of postoperative recurrence and controlling micro metastases. Unlike traditional cytotoxic agents, migration therapy is designed to “block cancer cells from leaving their original site or surviving in the bloodstream.”

In primary tumors, cancer cells initiate the key process of EMT to facilitate further dissemination and seeding. This process endows epithelial cells with mesenchymal characteristics, enhancing their migratory, invasive, and antiapoptotic capabilities. The regulation of core signaling pathways involved in EMT, such as the TGF-β/SMAD and Wnt/β-catenin signaling pathways, can weaken cancer cell migration and prevent local invasion at its origin. Certain traditional Chinese medicine compounds may inhibit EMT by modulating the p53/TGF-β1 and TGF-β1/SMAD pathways, thereby suppressing GC cell growth and metastasis. However, the specific active components of these formulations require further investigation. After undergoing EMT, cancer cells secrete proteases to degrade the ECM and induce cytoskeletal retraction, which paves the way for their migration. Simultaneously, cancer cells sense chemotactic signals and migrate toward specific directions. This review proposes a shift toward therapeutic strategies that block chemokine axes and inhibit MMPs. For example, Z-guggulsterone can partially target the CXCL12/CXCR4 axis, downregulate the CXCR4 protein and mRNA expression in triple-negative breast cancer (TNBC), and thereby inhibit tumor growth (118). Propranolol suppresses the expression of MMP-2, MMP-9, and EGFR, thereby inhibiting GC cell migration (119). Therapeutic strategies targeting MMPs can be found in the previous discussion and are not discussed here.

During the processes of dissemination and metabolism, cancer cells must rely on integrins to adhere to the vascular wall once they reach the vicinity of blood vessels. Cilengitide can inhibit CD51 (integrin αv) expression in CSCs *in vitro*, which suppressed cancer stem cell phenotypes, and validated the therapeutic efficacy of targeting integrins in patient-derived organoids and xenograft models (120). After cancer cells enter the bloodstream, they can be killed by the photodynamic activation of photosensitizers such as rose Bengal, which is achieved by energy transfer from green fluorescent protein (GFP) (121, 122). Jin Fukang can promote the recruitment of CTCs to NK cells, significantly enhancing the cytotoxic effect of NK cells on CTCs, and induce the apoptosis of CTCs via the Fas/FasL signaling pathway, ultimately inhibiting lung cancer metastasis (123). Although further research is needed before these technologies can be applied clinically, the experimental results demonstrate a reduction in CTCs and highlight the potential of strategies that target cancer cells during circulation.

Via strategies such as source inhibition, pathway disruption, key-step blockade, and in-transit clearance, migration therapy has demonstrated potential in reducing metastasis rates and delaying metastatic onset in some experimental studies. However, tumor metastasis is notably characterized by high plasticity and redundancy. Future research should focus on multitarget combination therapies, precise drug delivery, and the development of individualized treatment regimens that aim, to block tumor metastasis at its initiation and progression stages.

#### 5.3.2 Photodynamic therapy

An ideal treatment for advanced cancer should extend beyond merely the elimination of local lesions; it should also activate sustained systemic immune responses. To reduce the migration of tumor cells to distant organs, establishing long-term immune surveillance capabilities in patients while maximizing the preservation of normal tissue function is essential. PDT offers a potential strategy in this regard. PDT relies on interactions among a photosensitizer, a light source, and oxygen in the tissue (124). Upon laser irradiation, singlet oxygen and free radicals are generated, which exert antitumor effects via multiple mechanisms, including the induction of tumor cell apoptosis, necrosis, and autophagy, and the disruption of the tumor vasculature (125).

The translational application of PDT in GC-PM is currently grounded in robust preclinical evidence and preliminary clinical exploration has been initiated. In preclinical studies, PDT and chemotherapy therapy (CDX) were integrated into liposomes and demonstrated a dual-response of ROS/pH in the tumor microenvironment, which significantly increased the antitumor effect (126). Furthermore, a multifunctional liposomal delivery system has been developed and shown to synergistically induce immunogenic cell death and remodel the immunosuppressive TME, thereby providing a mechanistic basis for combining PDT with immunotherapy (127). However, this approach has several limitations GC has high specificity, and some patients have immune system deficiencies. Moreover, PDT induces immunogenic cell death to reverse immunosuppression. Therefore, the application of PDT in GC–PM still requires further analysis.

In terms of clinical exploration, no large-scale phase III clinical trials specifically investigating PDT for GC–PM have yet been reported, which highlights the challenges in translating this approach from the laboratory to the clinic. Existing small-scale clinical observations suggest that monotherapy with PDT offers limited long-term survival benefits for patients with advanced, high-burden GC–PM (only 6 surviving patients after 4 years of follow-up (128). This is primarily attributed to the inherent technical limitations of PDT, including insufficient light penetration depth, difficulties in achieving uniform light distribution within the peritoneal cavity, a and limited capacity to remodel the established immunosuppressive microenvironment. Ultimately, PDT may play a role as an adjunctive treatment strategy for immune regulation.

Consequently, the current research frontier has shifted from evaluating the efficacy of PDT monotherapy to exploring its value as a platform for combination therapies. For instance, studies have reported that RGK36 and RGK45 cells generate high levels of ROS when exposed to hyperthermia at 42 °C. Combining PDT with HIPEC chemotherapy may increase photosensitizer uptake and ROS production via thermal effects (129). Alternatively, the integration of PDT with HER2-targeted photosensitizer-antibody conjugates could achieve more selective tumor killing. For example, a study in which porphyrin was combined with trastuzumab for targeted PDT in HER2-positive GC demonstrated that the lethality of this combination on HER2-positive GC cells was twice that of trastuzumab alone (130). These combination strategies aim to overcome the limitations of PDT monotherapy and hold promise for positioning PDT as a precise adjuvant approach-either for eradicating microscopic residual disease after CRS or for reversing the local immunosuppressive microenvironment.

#### 5.3.3 Intratumoral immunotherapy

The aforementioned hyperthermia combined with ICIs treatment therapy can synergistically reverse the immunosuppressive TME through immune regulatory pathways that are activated alongside direct killing (131). Similarly, targeted intratumoral immunotherapy (ITI) involves the direct injection of immunostimulatory agents into tumor tissues to achieve high local concentrations, activate the TME, reverse immunosuppression, and induce systemic antitumor immunity. Compared with systemic ICIs, ITI significantly reduces systemic adverse effects via localized administration while simultaneously transforming the tumor itself into an “*in situ* vaccine” (132).

The core goal of ITI is to transform “cold” tumors into “hot” tumors via a series of meticulously designed mechanisms, including reversing local immunosuppression, creating an *in situ* vaccine effect, enhancing immune cell infiltration, inducing immune memory, and synergizing with systemic therapies. This approach aims to achieve robust and sustained antitumor immunity (133). The TME is saturated with various immunosuppressive factors, and the reversal of local immunosuppression is thus the starting point and foundation of the action of ITI. The inhibition of immune checkpoint molecules and inhibitory cytokines serves as a key therapeutic target in this process. The relationship between anti-PD-1 monospecific monoclonal antibodies (mAbs) and hyperprogressive disease (HPD) in GC patients and proposed that PD-1 blockade may promote the proliferation of highly suppressive PD-1^+^ eTreg cells in HPD, thereby inhibiting tumor immunity (134). Also, *Helicobacter pylori* can activate the TGF-β/Smad2/EMT pathway via HKDC to drive GC cell proliferation and metastasis (135). Combining Toll-like receptor 7 (TLR7) agonists with PD-L1 blockade activates innate immunity and promotes T-cell recruitment. This approach also shows significant potential in lung cancer cases with poor immune infiltration (136).

While breaking immunosuppression, ITI also triggers an “*in situ* vaccine” effect. A recent example is the use of doxycycline to induce Escherichia coli CN018, which controlled tumor growth and metastasis and conferred protective immunity against tumor rechallenge in mice (137). Furthermore, the oncolytic virus CF33 and its derivatives significantly reduced peritoneal tumors and ascites and prolonged survival in GC-PM models (138). These preclinical outcomes provide reference data for the development of peritoneal-targeted therapeutic strategies for GC-PM patients. ITI can specifically address the limitations of traditional cancer immunotherapy and systemic chemotherapy, such as drug toxicity, limited penetration into solid tumors, and potential induction of autoinflammatory responses. Moreover, by delivering drugs directly into the tumor, ITI increases local drug concentrations, optimizes dosing, reduces systemic toxicity, and improves patient prognosis.

## 6 Discussion

Research on GC-PM has shifted from a tumor cell-centric perspective toward an comprehensive understanding of the dynamic peritoneal microenvironment. This shift suggests that future treatment strategies should focus on reshaping the peritoneal ecosystem, changing it from a state that promotes metastasis to one that inhibits tumor spread. However, the clinical translation rate of drugs targeting the TME is relatively low. There are still two core obstacles. As follows:

First, tumor heterogeneity drives the paucity of reliable biomarkers and variable therapeutic responses (139). Although mutant allele tumor heterogeneity (MATH) (140) and single-cell sequencing techniques (141) have partially revealed tumor heterogeneity, the spatial heterogeneity between primary tumors and metastatic tumors, as well as among different metastatic sites, and their associations with existing clinical biomarkers (such as HER2, PD-L1, and CLDN18.2.) remain unclear. Microsatellite instability (MSI) and the two immune response dominant subtypes of Epstein-Barr virus (EBV) positivity are rare in advanced GC (142), further limiting the applicability of precise treatment.

Second, existing animal models cannot fully simulate the dynamics and immunological complexity of the host tumor microenvironment. PDX models cannot induce metastasis (143) and novel orthotopic models are operationally difficult and prone to leakage (144). Large animal models are more closely related to human physiology but have long modeling cycles and low repeatability (145). Moreover, genetically engineered mice, organoids, and humanized models have not yet fully replicated the immune and stromal interactions within the TME. An ideal model should possess high microenvironment fidelity and ability to simulate metastasis cascades, support real-time monitoring hold clinical relevance to increase the accuracy of new drug evaluation.

In response to these challenges, this review proposes to build the development of a “TME digital monitoring system” as a future research direction. This system integrates multiomics technologies such as single-cell sequencing, spatial omics, and liquid biopsy, combined with AI-driven big data analysis to form a dynamic monitoring platform (146). The aim is to capture molecular responses (such as early changes in ctDNA (147)) in real time, assess therapeutic efficacy prior to than imaging, and guide the analysis of drug resistance mechanisms. Although this system faces practical bottlenecks such as high technical costs and complex data analysis, it is expected to overcome the limitations of “static biopsy”, promote the efficient transformation of individualized treatment from mechanism research to clinical application, and ultimately achieve a substantive breakthrough of TME-targeted strategies in the treatment of GC-PM.

## 7 Conclusion

This review systematically introduces the three core mechanisms by through GC cells remodel the host TME: metabolic reprogramming, mechanical stress, and neural control. On this basis, we elaborate on the metastasis cascade of cancer cells in this tumor-promoting TME and propose an innovative sequential treatment strategy that first dismantles physical barriers, then blocks nutrient supply, and ultimately reverses immunosuppression. With respect to future development, this review highlights the two core obstacles (tumor heterogeneity and the existing animal models) currently faced by clinical translation and proposes a forward looking strategy for integrating a digital monitoring system. We believe that translating mechanistic insights into clinical applications could transform GC-PM from a clinical challenge into a manageable, thereby, providing new treatment options and hope for patients.

## Glossary

Ach: Acetylcholine
BCAA: Branched-chain amino acid
BPB: Blood- peritoneal barrier
CAFs: Cancer-associated fibroblasts
CCR1: Chemokine receptor 1
CDX: Chemotherapy
CPT1A: Carnitine palmitoyltransferase 1A
CRS: Cytoreductive surgery
CTCs: Circulating tumor cells
ctDNA: Circulating tumor DNA
DCs: Dendritic cells
DFS: Disease-free survival
EBV: Epstein-Barr virus
ECM: Extracellular matrix
EMT: Epithelial-mesenchymal transition
EVs: Extracellular vesicles
FAO: Fatty acid oxidation
GC: Gastric cancer
GFP: Green fluorescent protein
GM-CSF: Granulocyte-macrophage colony-stimulating factor
HCC: Hepatocellular carcinoma
HIPEC: Hyperthermic intraperitoneal chemotherapy
HPD: Hyperprogressive disease
IARC: International Agency for Research on Cancer
ICAM1: Intercellular adhesion molecule-1
ICIs: Immune checkpoint inhibitors
IFN-γ: Interferon-gamma
IFP: Interstitial fluid pressure
IL-6: Interleukin-6
IPP: Intraperitoneal pressure
ITI: Intratumoral immunotherapy
mAbs: Monospecific monoclonal antibodies
MATH: Mutant allele tumor heterogeneity
MDSCs: Myeloid-derived suppressor cells
MFS: Metastasis-free survival
MHC: Major histocompatibility complex
MMPs: Matrix metalloproteinases
MMT: Mesothelial-to-mesenchymal transition
MSI: Microsatellite instability
MST: Median survival time
OvCa: Ovarian cancer
OS: Overall survival
OXPHOS: Oxidative phosphorylation
PA: Palmitic acid
PDT: Photodynamic therapy
PDX: Patient-derived xenograft
PDGFC: Platelet-derived growth factor C
PFCCs: Peritoneal free cancer cells
PFS: Progression-free survival
PIPAC: Pressurized Intraperitoneal aerosol chemotherapy
PM: Peritoneal metastasis
PPP: Pentose phosphate pathway
PTX: Paclitaxel
RAM: Ramucirumab
SOAT1: Sterol o-acyltransferase1
TAMs: Tumor-associated macrophages
TANs: Tumor-associated neutrophils
TCA: Tricarboxylic acid
TF: Tissue factor
TGF-β: Transforming growth factor-beta
TLR7: Toll-like receptor 7
TME: tumor microenvironment
TNBC: Triple-negative breast cancer
Tregs: T cells
VEGF: Vascular endothelial growth factor
WAT: White adipose tissue
WIRA: water-filtered infrared A radiation.
